# Cytotoxic and Apoptotic Activities of Methanolic Subfractions of* Scrophularia oxysepala* against Human Breast Cancer Cell Line

**DOI:** 10.1155/2016/8540640

**Published:** 2016-02-29

**Authors:** Mona Orangi, Ardalan Pasdaran, Dariush Shanehbandi, Tohid Kazemi, Bahman Yousefi, Behnaz-Alsadat Hosseini, Behzad Baradaran

**Affiliations:** ^1^Immunology Research Center, Tabriz University of Medical Sciences, Tabriz, Iran; ^2^Tabriz University of Medical Sciences, International Branch of Aras, Tabriz, Iran; ^3^Medical Plant Processing Research Center, Shiraz University of Medical Sciences, Shiraz, Iran; ^4^Department of Pharmacognosy, Faculty of Pharmacy, Guilan University of Medical Sciences, Rasht, Iran; ^5^Department of Biochemistry and Clinical Laboratories, Faculty of Medicine, Tabriz University of Medical Sciences, Tabriz, Iran

## Abstract

Herbs have played a positive role in medicine for thousands of years. In the current study, we investigated the cytotoxicity effects of* Scrophularia oxysepala* methanolic subfractions and the underlying mechanism responsible for cell death in human breast carcinoma (MCF-7 cells) and mouse fibrosarcoma (WEHI-164 cells). From 60% and 80% methanolic fractions, four subfractions (Fa, Fb, Fc, and Fd), yielded from size exclusion by Sephadex-LH20 column chromatography, were chosen. MTT assay revealed that all subfractions significantly reduced cell viability after 24 h and 36 h in a dose-dependent manner; it is worth noting that Fa and Fb subfractions had the highest cytotoxicity, with IC_50_ values of 52.9 and 61.2 *μ*g/mL in MCF-7 at 24 h, respectively. ELISA, TUNEL, and DNA fragmentation assay revealed that antiproliferative effects of all subfractions were associated with apoptosis on cancer cells, without any significant effect on L929 normal cells. qRT-PCR data showed that, after 24 h treatment with IC_50_ concentrations of the subfractions, caspase-3 expression was increased in cancer cells while the expression of Bcl-2 was decreased.* S. oxysepala* methanolic subfractions induce apoptosis in MCF-7 and WEHI-164 cells and could be considered as a source of natural anticancer agents.

## 1. Introduction

Cancer is one of the most prevalent diseases in the world; in the United States only, 1,658,370 new cancer cases and 589,430 cancer deaths have been estimated in 2015. Breast carcinoma is the leading cause of cancer-related mortality in women, which now represents one in four of all cancers in women [1, 2]. Natural products have played a substantial role in the treatment of disease for thousands of years. Moreover, more than 60% of all drugs are derived from natural products. For instance, vinblastine, vincristine, camptothecin derivatives, topotecan, irinotecan, etoposide, epipodophyllotoxin, and paclitaxel are plant derived anticancer agents [3–6].

Scrophulariaceae are a family of annual and perennial herbs, with 3000 species and more than 200 genera, widely distributed around the world, notably in Asia and North America [7]. The therapeutic effect of different* Scrophularia* species is observed in scabies, eczema, psoriasis, inflammatory diseases, and tumors. Iridoids and phenylpropanoids classes of compounds are probably responsible for the aforementioned therapeutic benefits [8–12]. The cytotoxic effect of one of these species named* S. oxysepala* was assessed in some cancer cells, and it was unveiled that* S. oxysepala* induces apoptosis in the mentioned cells [13]. Apoptosis is a key cellular process and is a target for development of new anticancer therapeutics [14].

Apoptosis is the major programmed cell death mechanism for removing unwanted and detrimental cells in a silent manner during embryonic development, tissue homeostasis, and immune regulation. In addition, apoptosis results from a collapse of the cellular infrastructure through internal proteolytic digestion by some enzymes named caspases, which leads to cytoskeletal disintegration, metabolic derangement, and genomic fragmentation [15–17]. Caspases can be activated through two so-called extrinsic and intrinsic or mitochondrial pathways. The extrinsic pathway involves the activation of caspase-8 through binding of an extracellular death ligand, while the intrinsic pathway is mediated by mitochondria in response to massive intracellular death stimuli such as oncogene activation and DNA damage. In the intrinsic pathway, such stimuli trigger the release of proapoptotic molecules like cytochrome c from mitochondria that spark the subsequent activation of caspase-9 and also suppress the antiapoptotic Bcl-2 protein. Both the apoptotic pathways come together on the same terminal named execution pathway, which is initiated by activation of caspase-3 and terminated by cell death [14, 18–20].

Since the prior research has shown that* S. oxysepala* extract induces apoptosis in MCF-7 breast cancer cells [13], in the current study, we aimed to investigate the cytotoxic and apoptotic effects of the methanolic fractions of* S. oxysepala* on MCF-7 and WEHI-164 more thoroughly to access the active anticancer compounds of the extract. WEHI-164, the mouse fibrosarcoma cell, was chosen to confirm the aforementioned effect with the aim of carrying out the ensuing* in vivo* researches.

## 2. Material and Method 

### 2.1. Preparation of Subfractions

*S. oxysepala* was gathered from Gharedagh Mountain, 30 kilometers to Kaleybar, during flowering period. A voucher specimen (2821) has been deposited at the Herbarium of the Researches Center for Agriculture and Natural Resources, East Azerbaijan, Iran.

Air-dried and powdered aerial parts of* S. oxysepala* (1800 g) were extracted with methanol using Soxhlet apparatus. The plant extracts were concentrated by rotary evaporator in 45°C (Heidolph, Germany) under reduced pressure to obtain powder or viscous mass. Eight grams of methanolic extract was fractionated by solid phase extraction (SPE) method on a Sep-Pak (10 g) C_18_ cartridge using a step gradient of MeOH : water mixture (10 : 90, 20 : 80, 40 : 60, 60 : 40, 80 : 20, and 100 : 0). Two grams of the 60% and 80% fractions was dissolved in minimum probable amount of methanol and loaded on Sephadex-LH20 column using isocratic (CH_2_Cl_2_-MeOH, 1 : 1) elution which yielded twenty subfractions (F). Then, these subfractions were intermingled with similar pattern resulting from thin layer chromatography using chloroform : methanol system (7 : 3). Afterward, a pilot study is carried out for all subfractions and finally, four subfractions (Fa, Fb, Fc, and Fd) were chosen and assessed for their impacts. The amounts of Fa, Fb, Fc, and Fd subfractions were 68, 85, 60, and 53 mg, respectively.

### 2.2. Cell Culture

Human breast cancer cell line MCF-7, mouse fibrosarcoma cell line WEHI-164, and mouse normal control cell line L929 were obtained from the Iranian National Cell Bank (Pasteur Institute, Tehran, Iran). Cells were maintained in a humidified water-jacked incubator with 5% CO_2_ at 37°C in RPMI-1640 supplemented with 10% fetal bovine serum (FBS), penicillin (100 U/mL), and streptomycin (100 *μ*g/mL) (all purchased from Sigma, Germany).

### 2.3. MTT Assay

Cytotoxicity of the methanolic subfractions of* S. oxysepala* was evaluated using the MTT assay, which is based on the ability of viable cells to metabolize yellow tetrazolium salt MTT to purple formazan crystals by mitochondrial dehydrogenases. Briefly, cells were seeded at a density of 15000 per well in 96-well plates; subsequently, after 24 h incubation, they were treated with various concentrations (0–300 *μ*g/mL) of the aforementioned subfractions for 24 h and 36 h. The untreated well was considered as a negative control. Afterward, the suspended medium was thrown away and 20 *μ*L of 5 mg/mL MTT solution was added to each well and further incubated for 4 h at 37°C. Subsequently, the whole suspended medium was discarded from each well before adding 200 *μ*L DMSO and 50 *μ*L Sorenson buffer. In order to complete dissolution, the plate was incubated for 30 min with gentle shaking for 5 min. The cytotoxic effects of the subfractions were monitored by measuring the absorbance of each well at 570 nm (Awareness Technology, USA).

### 2.4. Cell Death Detection

The Cell Death Detection ELISA Kit (Roche Diagnostic GmbH, Germany) was used to detect apoptosis and necrosis in cells treated with the subfractions, according to the manufacturer's protocol. Firstly, cells (1 × 10^4^) were seeded in 96-well plates; after 24 h of treatment with the same concentration of subfractions the supernatants and lysate of cells were extracted and incubated in the microtiter plate modules coated with streptavidin. Subsequently, a mixture of anti-histone-biotin and peroxidase-conjugated anti-DNA antibody was used for the detection of immobilized histone/DNA fragments followed by color development with ABTS substrate for peroxidase. The results were analyzed spectrophotometrically using an ELISA plate reader at 405 nm.

### 2.5. DNA Fragmentation

DNA fragmentation which occurred in apoptosis was analyzed by agarose gel electrophoresis. Briefly, cells (7 × 10^5^ cells) were exposed to the aforementioned subfractions for 24 h and were gently scraped. Then, the extraction and sedimentation of DNA were performed by the proteinase K method and cold isopropanol, respectively (Cinnagen, Iran). Finally, 10 *µ*L of the DNA extract was loaded to 1.8% of agarose gel electrophoresis, stained with safe stain*™* (Cinnagen, Iran), observed under UV light.

### 2.6. TUNEL Assay

The TUNEL (terminal dUTP nick end-labeling) method is very sensitive and widely used to measure DNA fragmentation, which occurred in apoptosis. The principle of the assay is that endonuclease-generated DNA breaks are enzymatically labeled by terminal transferase with UTP derivatives coupled to biotin that can be detected in an immunoperoxidase reaction. The test was carried out according to the protocol of In Situ Cell Death Detection Kit POD (Roche Diagnostics GmbH, Germany). After subculture and treatment, the cells were fixed by 4% paraformaldehyde in PBS (pH 7.4) for 1 h at room temperature and rinsed with PBS. Then, blocking solution (3% H_2_O_2_ in methanol) was added and incubated for 10 min at the same temperature. The cells were washed and thereupon incubated in permeabilisation solution (0.1% Triton X-100 in 0.1% sodium citrate) for 2 min on ice. Subsequently, the slides were rinsed twice and 50 *µ*L of the reaction mixture, containing TdT enzyme and nucleotide, was added to the cells and incubated at 37°C for 1 h. After rinsing three times with PBS, the slides were incubated at 37°C with 50 *µ*L converter-POD (streptavidin HRP solution) for 30 min and rinsed three times with PBS. Finally, the cells were incubated with DAB substrate (Sigma, Germany) until a light brown background developed, and the stained cells were immediately observed under microscope.

### 2.7. Real Time PCR

The mRNA expression levels of widely established apoptotic and antiapoptotic related genes, caspase-3 and Bcl-2, were performed using quantitative reverse transcriptase-polymerase chain reaction (qRT-PCR). After subculture and treatment, total cellular RNA was isolated from the untreated and treated cells using an RNX PLUS Kit (Cinnagen, Iran) according to the manufacturer's protocol. Then the quality and quantity of isolated RNA were evaluated by a NANODROP 2000c spectrophotometer (Thermo Scientific, USA). Subsequently, the RNA was reverse transcribed into cDNA and used as the template for PCR amplification using a reverse transcriptase kit (Thermo Scientific, USA). Quantitative RT-PCR (qRT-PCR) was performed by the Corbett Rotor-Gene 6000 system (Corbett Life Science, Australia). PCR was carried out in a final volume of 20 *μ*L reaction system containing 0.2 *μ*M of each primer (Table 1), 10 *μ*L of SYBR green reagent (RR820L Takara Bio, Japan), 1 *μ*L of cDNA template, and 8.6 *μ*L of nuclease-free water.

The PCR cycling was carried out by initial denaturation step at 95°C for 3 min followed by 45 cycles at 95°C for 10 seconds, 58°C for 30 seconds, and 72°C for 20 seconds. Relative mRNA expression was measured by the 2^−(ΔΔCT)^ method, using *β*-actin and GAPDH as reference genes [21].

### 2.8. Statistical Analysis

All the data represented in this study are expressed as means ± SD. The experiments were assayed in triplicate (*n* = 3). Analysis of variance (ANOVA) followed by a two-tailed unpaired *t*-test was used to determine the significant differences between groups and *p* value <0.05 was considered significant. All statistical analyses were conducted using GraphPad Prism 6.01 software (GraphPad Software Inc., San Diego, CA, USA).

## 3. Result 

### 3.1. The Cytotoxicity of* S. oxysepala* Methanolic Subfractions

**Figure 1:**
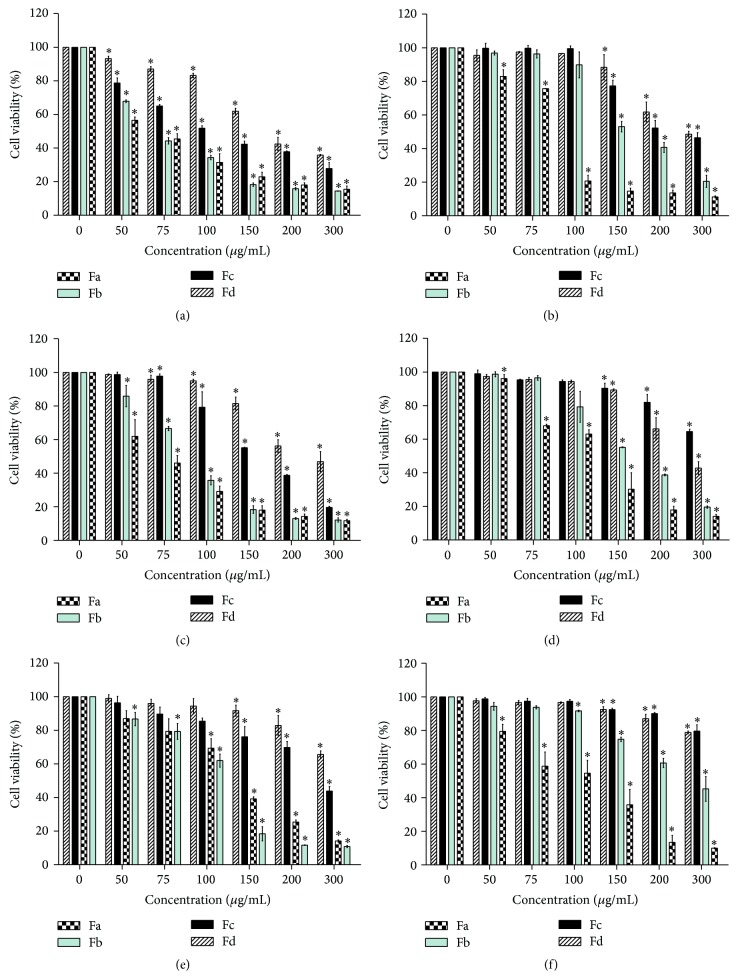
Effects of* S. oxysepala* methanolic subfractions on viability of the cells after 24 h and 36 h, using MTT assay. (a) MCF-7 in 24 h; (b) MCF-7 in 36 h; (c) WEHI-164 in 24 h; (d) WEHI-164 in 36 h; (e) L929 in 24 h; and (f) L929 in 36 h. Values are presented as means (*n* = 3) ± SE. *∗* represents significant statistical difference (*p* < 0.05) relative to the blank control.

The effect of* S. oxysepala* subfractions on the viability of MCF-7 and WHEHI-164 cells was assessed using MTT assay. All tested subfractions caused a significant dose-dependent reduction in cell viability (relative to the blank control) after 24 h and 36 h (Figure 1).  Table 2 shows the IC_50_ (50% inhibitory concentrations) values of the subfractions on the cells. A more meticulous analysis of data unveils that, in comparison to other subfractions, the Fa and Fb subfractions had the highest cytotoxicity on the cells (*p* < 0.05). Moreover, the cytotoxicity of all the subfractions on the MCF-7 and WEHI-164 was substantially higher than on L929 as a normal cell line (*p* < 0.05). However, the results show that the cytotoxic effects of all fractions reduced after 36 h incubation compared with 24 h incubation in all cell lines. To confirm the higher cytotoxic effect of the subfractions after 24 h incubation in comparison with 36 h incubation, we repeated the test three times and the result was the same.

### 3.2. Assessment of Necrosis and Apoptosis

**Figure 2:**
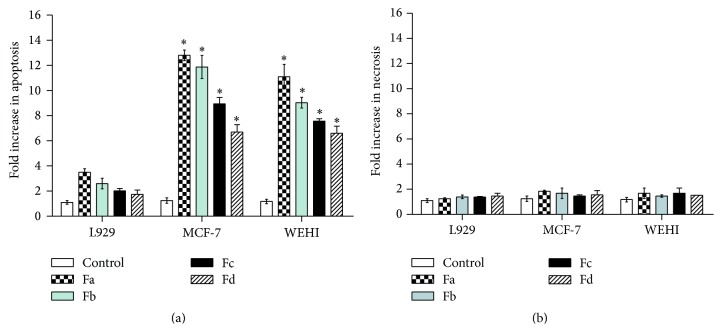
The apoptotic (a) and necrotic (b) effect of* S. oxysepala* methanolic subfractions (75 *μ*g/mL) on MCF-7, WEHI-164, and L929 cells after 24 h detected by cell death ELISA assay. Data are presented as the fold increase in apoptosis and expressed as means ± SE. *∗* represents significant difference (*p* < 0.05) relative to control.

Cell death detection ELISA kit was used to investigate whether the cytotoxicity of subfractions is due to apoptosis or necrosis. All subfractions caused a significant increase in apoptosis rate in comparison with blank controls (*p* < 0.05). The cell death ELISA indicated 13-, 12-, 9-, and 6-fold increase in apoptosis in MCF-7 cells treated with Fa, Fb, Fc, and Fd versus untreated cells, respectively; moreover, Fa, Fb, Fc, and Fd treated WEHI-164 cell showed 11-, 9-, 7-, and 6-fold increase in apoptosis relative to control, respectively. However, the subfractions induced less apoptosis in L929 normal cells compared to the MCF-7 and WEHI-164 (Figure 2(a)). In addition, the number of necrotic cells in all cell line supernatants was determined; no statistically significant differences were found as compared to controls (Figure 2(b)).

### 3.3. Induction of Apoptosis by the Methanolic Subfractions of* S. oxysepala*

Apoptosis was assessed by TUNEL and DNA fragmentation assays which indicated the presence of DNA fragmentation as a biological hallmark of apoptosis.

**Figure 3:**
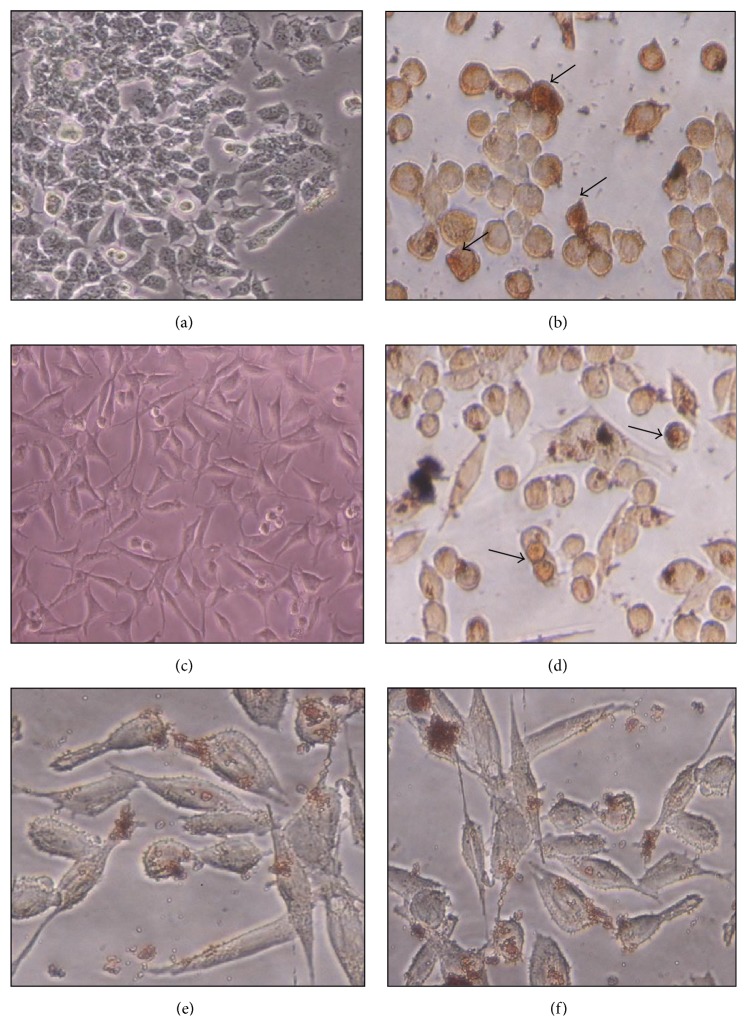
Dark stained nuclei (arrows) of the MCF-7 and WEHI-164 cells were observed after cell treatment with the methanolic subfraction (Fa) of* S. oxysepala*, whereas no stained nuclei were noted in the untreated (control) cells and treated L929 cell. (a) MCF-7 control, (b) treated MCF-7, (c) WEHI-164 control, (d) treated WEHI-164, (e) L929 control, and (f) treated L929. Arrows indicate representative apoptotic cells. (a)–(f): 200x magnification.

The results of TUNEL assay are shown in Figure 3; the cells treated with the subfractions produced dark brown stained nuclei, while none of the cell nuclei was stained in the untreated cells (negative control cells). Moreover, it is determined that IC_50_ concentrations of all subfractions created stained nuclei in MCF-7 and WEHI-164 cells; by contrast, there were no significant stained nuclei in treated L929 cell (normal cell line) upon treatment with the same dose.

**Figure 4:**
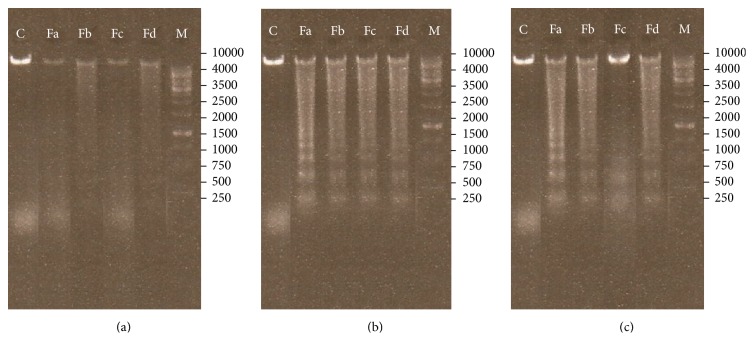
Effects of the methanolic subfractions of* S. oxysepala* on DNA fragmentation in the cells. (a) L929, (b) MCF-7, and (c) WEHI-164 (left to right: control, Fa, Fb, Fc, and Fd subfractions). Control = untreated cell, M = size marker.

The DNA fragmentation assay was performed in a 1.8% agarose gel after exposing the cancer cells to IC_50_ concentrations of the subfractions for 24 h. As shown in Figure 4, fragmented DNA was clearly observed in cancer cells whereas control cells did not provide ladders.

### 3.4. Expression of Apoptotic and Antiapoptotic Genes in the Cells Treated by Methanolic Subfractions of* S. oxysepala*

**Figure 5:**
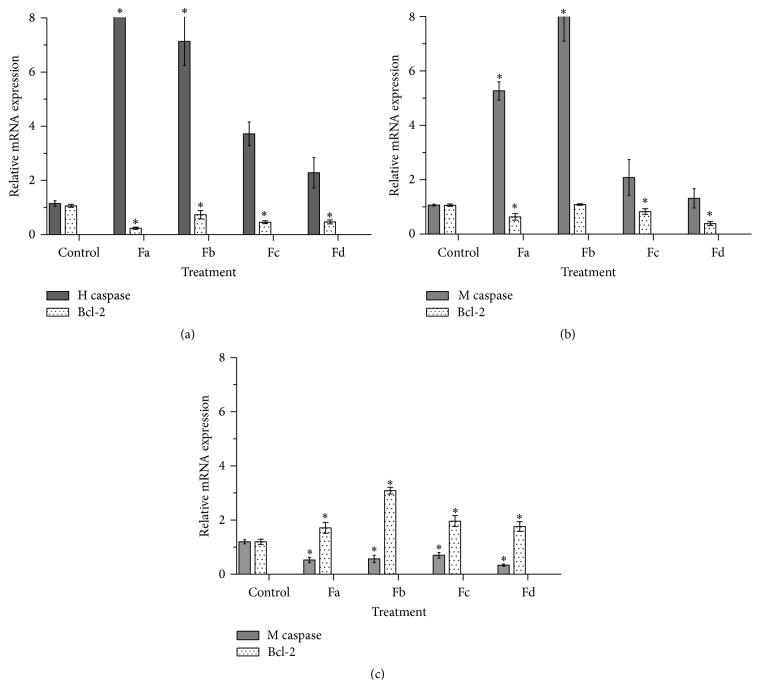
Effect of* S. oxysepala* methanolic subfractions on (a) caspase-3 and (c) Bcl-2 mRNA expression in MCF-7 (a), WEHI-164 (b), and L929 (c) cells at 24 h. Relative expression was acquired by qRT-PCR using 2^(−ΔΔCT)^ method. The results are presented as mean ± SD (*n* = 3); ^*∗*^ *p* < 0.05 versus control.

In order to determine the expression level of apoptotic and antiapoptotic genes in treated cells, the mRNA levels of caspase-3 and Bcl-2 were evaluated by qRT-PCR. After 24 h treatment with IC_50_ concentrations of the subfractions, caspase-3 expression was induced in tumor cells, with 11.31-, 7.21-, 3.94-, and 2.62-fold increase in MCF-7 cells and 5.17-, 8.34-, 2.04-, and 1.24-fold increase in WEHI-164 cells treated with Fa, Fb, Fc, and Fd, respectively (compared to blank controls); by contrast, the expression level of Bcl-2 mRNA declined in both cell lines (Figures 5(a) and 5(b)). However, the expression of these genes in treated L929 cells showed a different pattern; the expression of caspase-3 decreased while the cells treated with Fa, Fb, Fc, and Fd caused 1.71-, 3.07-, 1.98-, and 1.79-fold increase in the expression level of Bcl-2 mRNA (Figure 5(c)).

## 4. Discussion

Since the therapeutic and anticancer effects of different* Scrophularia* species have been investigated in many studies [7–10], coupled with the findings of the previous study which revealed that* S. oxysepala* extract can induce apoptosis in the breast cancer cell line MCF-7 [22], in the present study, we evaluated the cytotoxic and apoptotic effects of the methanolic subfractions of* S. oxysepala* on MCF-7 and WEHI-164 cell lines to potentially obtain the active subfraction of the extract. From 60% and 80% methanolic fractions, four subfractions (Fa, Fb, Fc, and Fd), yielded from size exclusion by Sephadex-LH20 column chromatography and inhibiting the growth of the mentioned cells, were chosen. From those, Fa and Fb had the highest cytotoxicity, with IC_50_ values of 52.9 and 61.2 *μ*g/mL in MCF-7 at 24 h, respectively (Table 2). In a previous study as noted above [13, 22], the IC_50_ value of the methanolic extract in the same cell line at 24 h was 180.5 *μ*g/mL; therefore, it could be concluded that the most active compounds of the plant may be in Fa and Fb subfractions. Other* Scrophularia* species have also been reported to have cytotoxic effects against various tumor cells. For example, Giessrigl et al. have shown that the methanol extracts of* S. floribunda*,* S. lucida*,* S. peregrine*,* S. pinardii*, and* S. libanotica* inhibited cell growth, with IC_50_ values of 0.5, 0.4, 3.7, 0.9, and 0.9 mg/mL in HL-60 promyelocytic leukemia cells at 72 h, respectively [7]. Shen et al. reported the cytotoxic effect of* S. ningpoensis* in three different Colo 38, SK-Mel-28, and MRI-22 melanoma cell lines [23].

Most anticancer therapeutics relies on induction of apoptosis for inducing cell death in cancer cells and eradication of tumors [24, 25]. Therefore, to distinguish the type of cell death, TUNEL and DNA fragmentation assays were performed. Both assays demonstrated that the methanolic subfractions induce apoptosis in cancer cells without any significant effect on normal L929 cells as control (Figures 3 and 4). These tests are common in the literature for probing apoptosis in response to natural products. For example, in one study, Machana et al. performed DNA fragmentation assay to show apoptosis in HepG2 cells treated with the extracts of five plants [26]. Reddivari et al. have also reported the apoptotic effects of the potato extract on PC-3 and LNCaP prostate cancer cells by TUNEL assay and ELISA [27]. We employed ELISA to determine the level of apoptosis and though all subfractions caused a significant fold increase in apoptosis, as expected, Fa had the most prominent effect (Figure 2).

In the current study, the mRNA expression levels of two apoptotic and antiapoptotic genes, namely, caspase-3 and Bcl-2, were investigated in cells treated with the subfractions.  Figure 5 illustrates that the subfractions caused an increased expression of caspase-3 mRNA in MCF-7 and WEHI-164. However, there were no substantial changes in the expression of caspase-3 in L929 normal cells. Furthermore, the expression of Bcl-2 decreased in cancer cell lines. Evaluation of caspase and Bcl-2 expression is a common approach used for analysis of apoptosis upon treatment with compounds. For example, downregulation of Bcl-2 has been already reported in MCF-7 and WEHI cells after induction of apoptosis by other compounds [28, 29].

**Figure 6:**
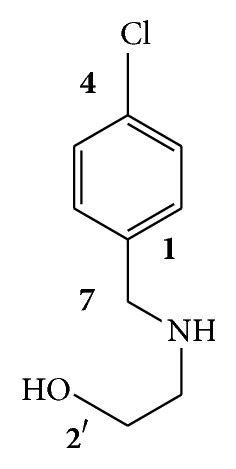
Chemical structure of isolated compound from Fa subfraction.

A large number of anticancer compounds, which are available in the market, have been isolated from plants [30]. Some cytotoxic compounds have been isolated from* Scrophularia* species, such as iridoid glycosides [31–34]. Iridoid glycosides and their hydrolysed products have shown anticancer activity against cervical carcinoma Hela, gastric carcinoma MNK-45, and myeloid leukemia K562 cell lines [35–37]. Based on our pharmacognostic investigation, some of these iridoid glycosides such as scropolioside D and harpagoside B have been isolated from our methanolic subfractions, and also 2-(4-chlorobenzyl amino) ethanol has been isolated from Fa subfraction which had significant cytotoxic effect among other fractions (Figure 6) (not reported yet), and we presume that these compounds might be the active anticancer compounds within the subfractions; however, they should be explored in further research. Furthermore, upon confirmation of pure anticancer compounds from the fractions, we plan to investigate the* in vitro* and* in vivo* anticancer potential of the fractions.

## 5. Conclusion

Based on the results of this study, the methanolic subfractions of* S. oxysepala* induce apoptosis in MCF-7 and WEHI-164 cells in a dose-dependent manner and these fractions can thus be considered as a source of anticancer compounds. Furthermore, these subfractions are not cytotoxic against the L929 normal cell line, which is another advantage.

## Figures and Tables

**Table 1:** The sequence of primers used in RT-PCR.

Primer	Sequence(s) 5′-3′
*β*-actin	
Forward	TCCCTGGAGAAGAGCTACG
Reverse	GTAGTTTCGTGGATGCCACA
M caspase-3 (*Mus musculus* caspase-3)	
Forward	TGTCATCTCGCTCTGGTACG
Reverse	AAATGACCCCTTCATCACCA
Bcl-2 (human and mouse Bcl-2)	
Forward	CCTGGGATGACTGAGTACC
Reverse	GAGACAGCCAGGAGAAATCA
H caspase-3 (human caspase-3)	
Forward	ATGGTTTGAGCCTGAGCAGA
Reverse	GGCAGCATCATCCACACATAC
M GAPDH (*Mus musculus* glyceraldehyde-3-phosphate dehydrogenase)	
Forward	CCTCGTCCCGTAGACAAAA
Reverse	AATCTCCACTTTGCCACTG

**Table 2:** IC_50_ concentrations (*µ*g/mL) of methanolic subfractions of* S. oxysepala* on MCF-7, WEHI-164, and L929 cell lines after 24 and 36 h incubation.

	Subfractions	Fa	Fb	Fc	Fd
24 h	MCF-7	52.9	61.2	80.4	141
	WEHI-164	59.4	81.8	146.4	192.4
	L929	126	106	>300	>300

36 h	MCF-7	83.1	145.4	182.5	222.6
	WEHI-164	102.6	147.3	>300	229.5
	L929	113	175	>300	>300
